# Understanding Hip Surgical Approaches: A Review With Clinical and Imaging Correlation

**DOI:** 10.7759/cureus.86724

**Published:** 2025-06-25

**Authors:** Juan Pablo Munoz, Ignacio Espinoza, Sebastian Figueroa

**Affiliations:** 1 Skeletal Radiology, Clinica MEDS, Santiago, CHL

**Keywords:** arthroplasty, arthroscopy, clinical correlation, hip approaches, postoperative imaging

## Abstract

Hip surgery is evolving rapidly, with an increasing diversity of techniques across arthroplasty and arthroscopy. This diversity presents challenges not only to radiologists interpreting postoperative imaging but also to general clinicians assessing surgical outcomes. A clear understanding of the various surgical approaches, their anatomical pathways, and associated complications is essential for multidisciplinary care. This review summarizes the key surgical access routes used in hip procedures, outlines their technical features, and highlights relevant postoperative findings, including potential nerve and soft tissue complications. The goal is to provide clinicians, surgeons, and imaging specialists with a unified understanding of modern hip surgical access and its implications for patient evaluation.

## Introduction and background

Since the first documented total hip arthroplasty (THA) by Sir John Charnley in 1960 [1], various surgical approaches have been developed for hip procedures, including THA, osteotomies, fracture fixation, and oncologic resections. Variations in technique among surgeons and institutions make it essential to understand both standard and historical methods for accurate interpretation of postoperative findings.

Advances in arthroscopy introduced hip procedures in the 20th century, initially limited to the peripheral compartment and later expanding to the central compartment. Today, femoroplasty, labral repair, and acetabuloplasty are the most common hip arthroscopic procedures worldwide [2].

Bridging the knowledge gap between surgical access and imaging is crucial, as evolving techniques complicate the identification of normal and abnormal postoperative appearances. A thorough understanding of orthopedic procedures, ideally supported by surgical records, aids in accurate interpretation. While most literature focuses on implants and complications, limited data exist on the imaging findings of specific surgical approaches. Recognizing the technique used can be key to identifying complications and avoiding misinterpretation of expected postoperative changes.

This review provides an overview of commonly used hip surgical approaches, illustrating normal postoperative anatomy and selected abnormal findings specific to each approach. Information on procedure-specific complications is noted where relevant.

## Review

Posterior approach

**Figure 1:**
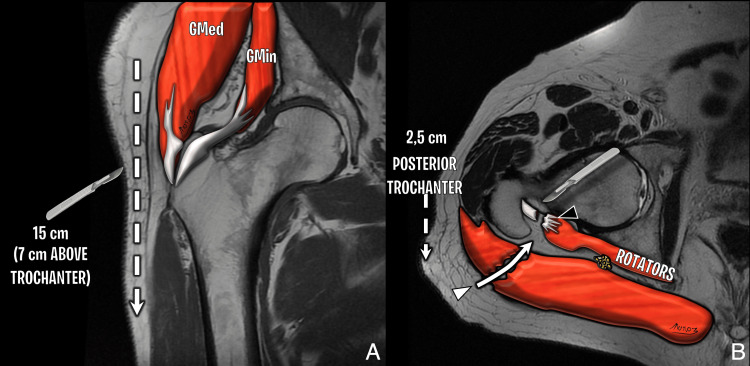
Illustration of posterior hip approach (A) Coronal T1-weighted image depicting the extent of the lateral skin incision. Gluteus minimus and medius tendons are preserved. (B) Axial T2-weighted diagram showing dissection of the lateral gluteus maximus near the gluteal tuberosity (white arrowhead) and tenotomy of the short external rotators, in this case the obturator internus–gemelli complex (black arrowhead). Image Credit: Original artwork by Juan Pablo Muñoz, corresponding author GMed: gluteus medius; GMin: gluteus minimus

**Figure 2:**
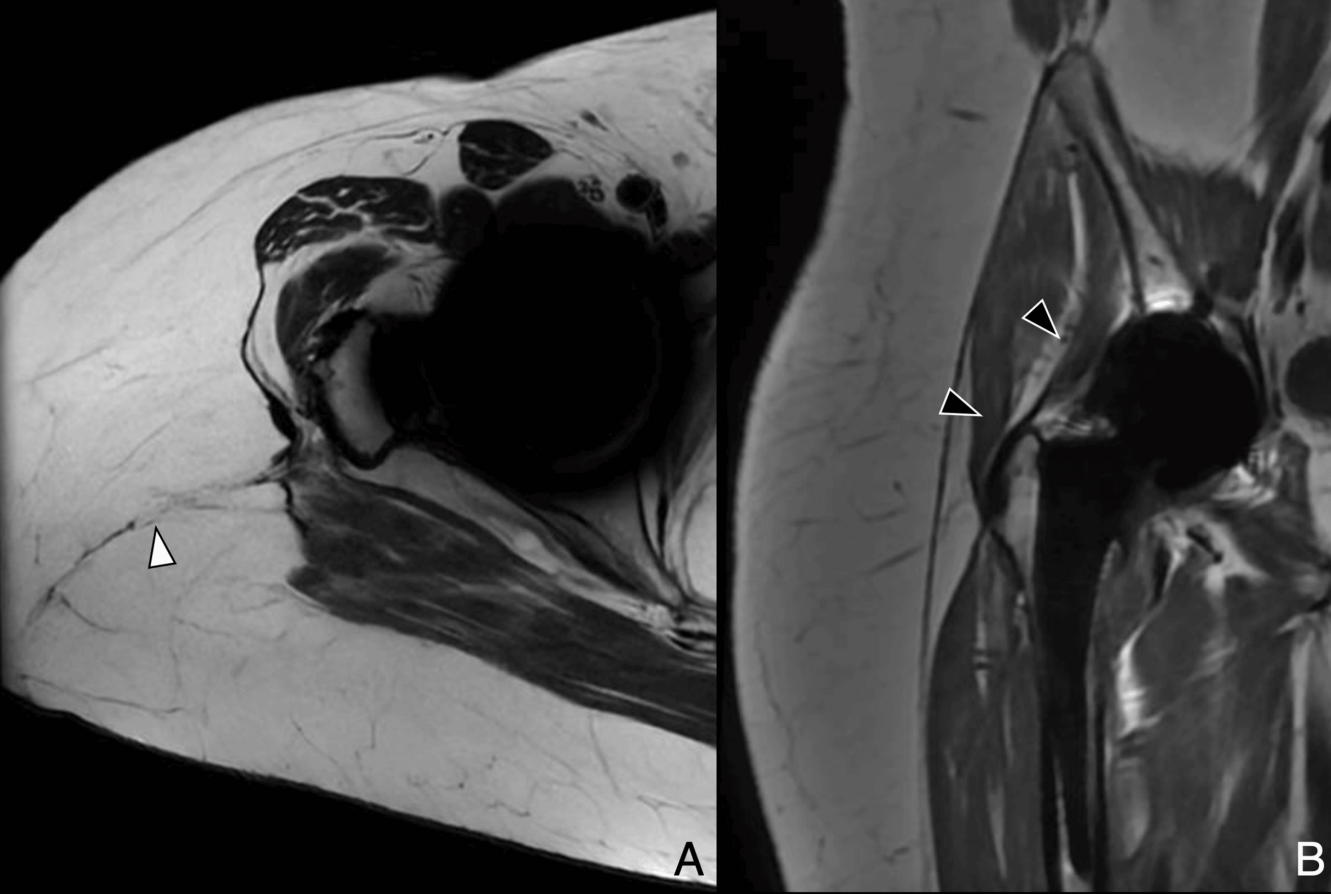
Posterior approach: postoperative MRI (A) Axial T2-weighted image showing hypointense scar in the subcutaneous tissue (white arrowhead). (B) Coronal MAVRIC (multi-acquisition variable resonance image combination) sequence demonstrates preserved abductor tendons (black arrowheads). Image Credit: Juan Pablo Muñoz, corresponding author

The posterior approach remains a widely used method for THA and has historically been favored for its excellent visualization of the acetabulum and femur. It involves a 10 cm incision extending from approximately 5 cm below the greater trochanter toward the posterior superior iliac spine. Access is gained through blunt dissection of the gluteus maximus, followed by tenotomy of the short external rotators and the piriformis tendon at their insertions. Preservation of the gluteus medius and minimus tendons allows for maintained abductor function. Postoperative imaging may reveal a scar within the gluteus maximus and signs of tenorrhaphy at the piriformis and obturator internus-gemelli complex. Piriformis atrophy is a frequent finding even with adequate repair (Figure 1, Figure 2) [3,4].

Recent comparative studies have demonstrated that posterior and anterior approaches yield statistically and clinically similar functional outcomes by three and 12 months postoperatively, as measured by both patient-reported and objective functional tests [5,6]. Although early subjective recovery may slightly favor the anterior approach in some series, objective measures such as the timed up and go (TUG) test, four-meter walk test (4MWT), and 30-second sit-to-stand (30STS) test demonstrate equivalent improvement between approaches at long-term follow-up [6]. Furthermore, the posterior approach continues to show a shorter operative time compared to anterior-based techniques, providing logistical advantages in certain surgical settings [6].

Dislocation has historically been cited as a potential disadvantage of the posterior approach. However, adherence to postoperative precautions and advancements in capsular repair techniques have significantly mitigated this risk [7-9]. In modern series, the difference in dislocation rates between posterior and anterior approaches has diminished, and when posterior precautions are followed, the rates appear comparable [6]. The posterior approach remains an excellent option in THA, especially when considering its familiarity among surgeons, reproducibility, and consistent clinical outcomes. Patient-specific factors, surgeon experience, and institutional protocols should guide the selection of the approach rather than perceived superiority based solely on approach-related differences [6].

Lateral approach

**Figure 3:**
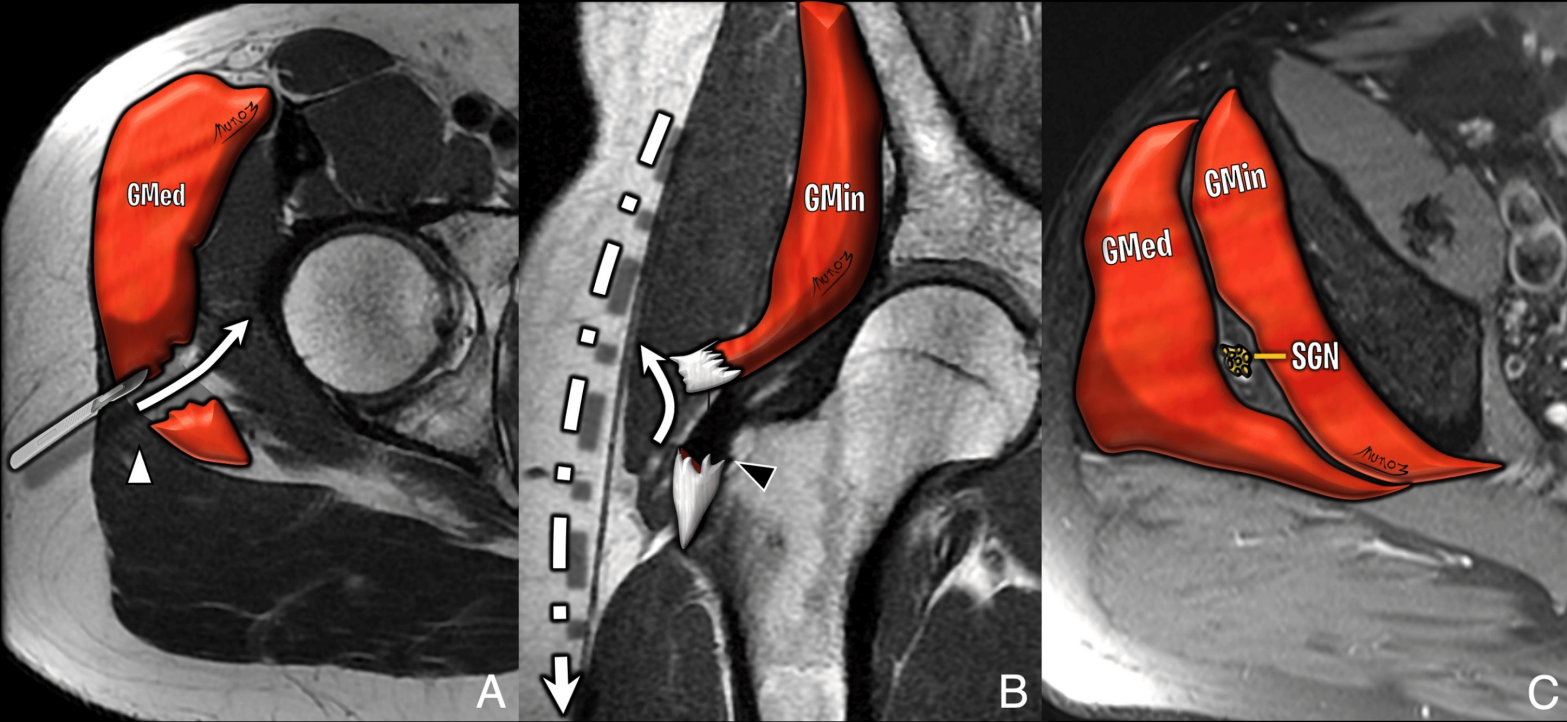
Illustration of lateral hip approach (A) Axial T2-weighted image showing gluteus medius split (white arrowhead). (B) Gluteus minimus tenotomy (black arrowhead) and skin incision extending above and below the greater trochanter. (C) SGN shown at risk in this approach. Image Credit: Original artwork by Juan Pablo Muñoz, corresponding author GMed: gluteus medius; GMin: gluteus minimus; SGN: superior gluteal nerve

**Figure 4:**
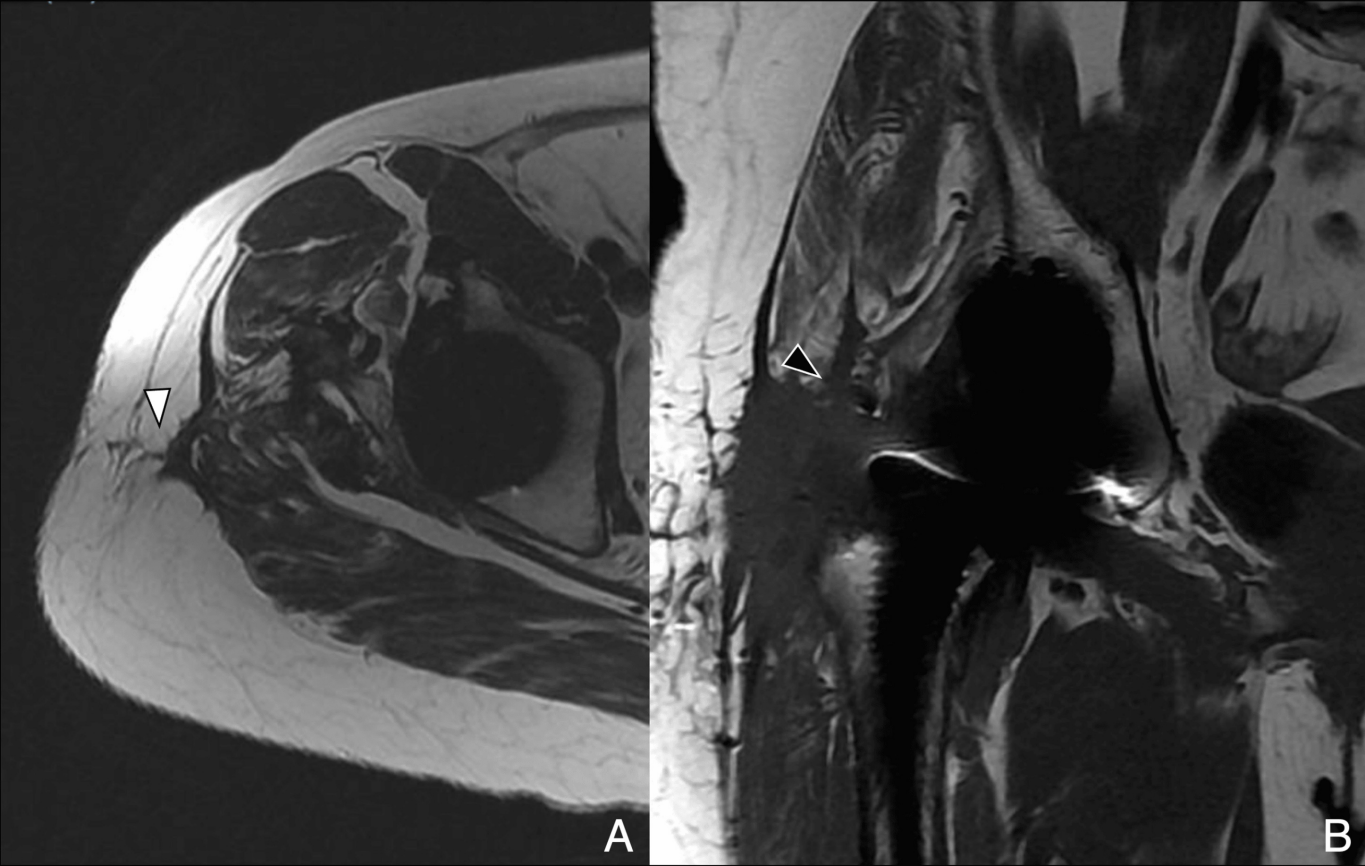
Lateral approach: postoperative MRI (A) Axial T2-weighted image shows subcutaneous scar tract (white arrowhead). (B) Coronal T1-weighted image shows a scar at the myotendinous junction of the gluteus minimus (black arrowhead) and scar along the gluteus medius dissection plane. Image Credit: Juan Pablo Muñoz, corresponding author

The lateral approach, first described by Hardinge in 1982, remains widely used in many centers, particularly in Europe [10]. It involves a 10 cm incision extending 5 cm above and below the greater trochanter. The gluteus medius is split between its anterior and middle thirds to access the gluteus minimus, which is then detached to expose the capsule [4]. Care must be taken not to extend the dissection more than 3-5 cm above the tip of the greater trochanter to avoid injury to the superior gluteal nerve (SGN). On postoperative imaging, abductor detachment or scarring may be noted. While the SGN is at risk during this approach, the lateral femoral cutaneous nerve (LFCN) is typically spared. MRI findings may demonstrate gluteal tendon disruption or muscle atrophy, important clues in the diagnosis of abductor insufficiency (Figure 3, Figure 4).

Despite the popularity of this approach, concerns remain regarding its potential for postoperative abductor dysfunction. Recent evidence using quantitative electromyography (QEMG) has demonstrated that up to 37.5% of patients show early EMG evidence of abductor muscle denervation six weeks after surgery, though the majority recover by 12 weeks postoperatively [11]. This dysfunction appears to result primarily from stretching of the SGN rather than direct transection, especially if dissection remains within the recommended “safe zone.” Residual weakness, when present, is generally mild and not associated with major functional impairment.

Functional outcomes following the lateral approach are generally good but may lag slightly compared to other techniques. A 2022 network meta-analysis of 63 randomized controlled trials found that the direct lateral approach (DLA) was associated with a smaller improvement in hip function scores compared with other approaches, including posterior and anterior techniques [6]. Moreover, DLA was associated with higher blood loss intraoperatively compared to minimally invasive approaches. Nevertheless, complication rates, including dislocation, fracture, and thromboembolism, were similar across all surgical approaches [6].

Clinically, persistent abductor weakness after the lateral approach may manifest as a positive Trendelenburg sign or limp, although this finding is multifactorial and may not always correlate with electrophysiological evidence of nerve injury [12]. Factors such as incomplete repair of the abductors, variations in surgical technique, and preoperative muscle status also play important roles. Overall, when technical refinements, such as limiting the extent of the gluteus medius split and careful handling of soft tissues, are respected, the lateral approach remains a reliable and effective method for hip arthroplasty.

Anterior approach

**Figure 5:**
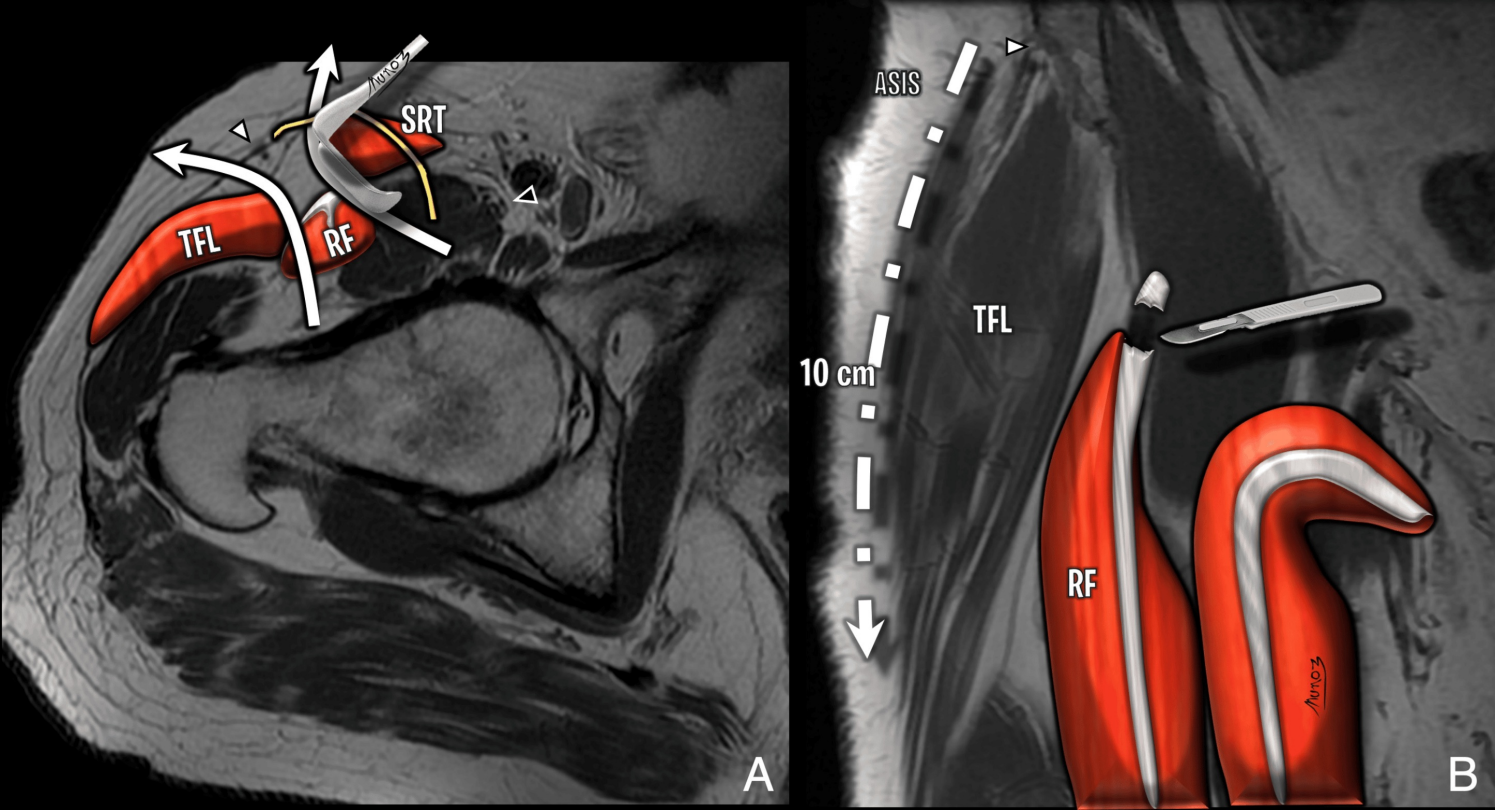
Illustration of the anterior approach (Smith-Petersen interval) (A) Axial T2-weighted diagram showing the interval between sartorius and tensor fascia lata (TFL), exposing the rectus femoris. Lateral femoral cutaneous nerve (white arrowhead); femoral nerve (black arrowhead). (B) Coronal T1-weighted diagram showing proximal detachment of the rectus femoris and the course of the lateral femoral cutaneous nerve (white arrowhead). Image Credit: Original artwork by Juan Pablo Muñoz, corresponding author TFL: tensor fascia lata; RF: rectus femoris; SRT: sartorius; ASIS: anterior superior iliac spine

**Figure 6:**
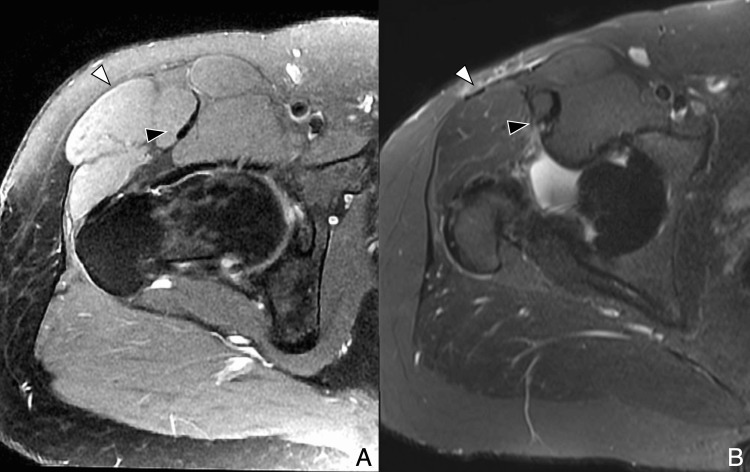
Anterior approach (Smith-Petersen interval): pre- and postoperative findings (A) Preoperative axial proton density fat-saturated (PDFS) image. (B) Postoperative axial short tau inversion recovery (STIR) with slice encoding for metal artifact correction (SEMAC). Postoperative thickening of TFL fascia (white arrow) and thickening of the rectus femoris tendon (black arrow) from partial release. Access is anterior to the TFL (distinct from the anterolateral Watson-Jones interval). Image Credit: Juan Pablo Muñoz, corresponding author

The anterior approach, first described by Smith-Peterson in the 1940s [13], is now preferred in many centers for hip arthroplasty due to faster gait restoration and lower dislocation rates [3,7]. Variants, such as muscle-sparing and tendon-detaching techniques, influence postoperative anatomy. The procedure involves a 10 cm incision near the anterior superior iliac spine, extending toward the lateral patella. The LFCN is isolated and retracted, and a working interval is developed between the sartorius and tensor fascia lata (TFL), known as the Smith-Peterson interval [6]. The rectus femoris tendon may be spared or detached. Potential complications include femoral nerve and LFCN injuries, both of which may manifest with characteristic imaging or clinical signs (Figure 5, Figure 6).

Direct Anterior Approach (DAA)

Compared to other techniques, the DAA is associated with specific advantages and disadvantages. According to a large 2022 network meta-analysis of 63 randomized controlled trials, the DAA demonstrated a statistically significant improvement in hip function scores compared with the direct lateral approach (mean difference: 4.04; 95% CI: 1.92-6.16) [6]. Short-term patient-reported outcomes, including lower early postoperative pain scores and faster mobilization, have been consistently superior in DAA patients compared to lateral or posterior approaches. Additionally, the DAA is associated with a reduced risk of dislocation, with large retrospective studies reporting rates as low as 0.23% [14].

However, the DAA presents unique technical challenges. It is associated with longer operative times compared to posterior and lateral approaches, particularly during the surgeon’s learning curve, and carries a higher risk of intraoperative femoral fractures (0-5.3%) [14]. The incidence of LFCN neurapraxia is also higher, although its functional impact appears minimal. Blood loss is greater than in minimally invasive lateral or posterior techniques, although the clinical significance remains debatable [6].

Importantly, while the DAA provides excellent early functional results, meta-analyses and long-term data suggest no clear superiority in implant survival compared to posterior or lateral approaches. As with all techniques, outcomes are heavily surgeon-dependent. Proper training, experience, and intraoperative fluoroscopic assistance are essential to achieve optimal results with the DAA.

Modified Anterior Approach

**Figure 7:**
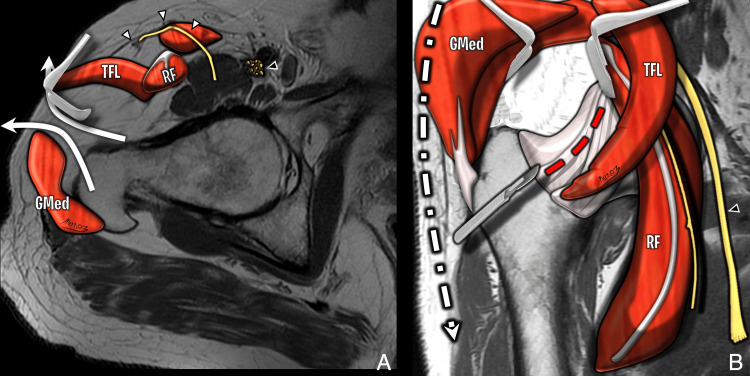
Illustration of the anterolateral muscle-sparing approach (Watson-Jones interval) (A) Axial T2-weighted diagram showing access between tensor fascia lata and gluteus medius. Lateral femoral cutaneous nerve (white arrowhead); femoral nerve (black arrowhead). (B) Coronal T1-weighted diagram showing access between gluteus medius, minimus, and tensor fascia lata (curved arrows). Nerves are displaced medially. Femoral nerve is protected if dissection remains lateral to sartorius. Image Credit: Original artwork by Juan Pablo Muñoz, corresponding author GMed: gluteus medius; TFL: tensor fascia lata; RF: rectus femoris

Several modifications of the classic DAA have been proposed to address its limitations, particularly concerning extensibility and femoral exposure. Modified anterior approaches, often using slightly extensile incisions or adjusted intermuscular planes, aim to facilitate femoral preparation and reduce intraoperative complications (Figure 7).

Cadaveric and clinical studies have shown that proximal and distal extensions of the modified anterior approach are feasible without significantly compromising neurovascular structures, provided careful technique is employed [14]. Techniques such as the subvastus extension help avoid injury to the lateral femoral circumflex artery branches and their motor branches to the vastus lateralis and vastus intermedius muscles.

Clinically, modified anterior approaches retain the muscle-sparing benefits of the DAA, with similar advantages in terms of early postoperative recovery, hospital stay reduction, and lower dislocation rates. Intraoperative fluoroscopy remains a valuable adjunct, facilitating accurate implant positioning [14]. However, technical demands remain high. Early mechanical complications, such as femoral stem malpositioning and undersizing, have been reported, particularly during the learning curve. Cementing quality, however, does not appear compromised compared to other approaches.

Concerns regarding wound healing, particularly in obese patients, have been raised, but infection and wound complication rates do not seem substantially different compared to posterior approaches when modern techniques are employed. Overall, the modified anterior approach offers a versatile alternative for surgeons familiar with the DAA, maintaining its muscle-sparing benefits while allowing improved access for challenging cases, such as revision surgeries or patients with difficult femoral anatomy.

Hip arthroscopy

Hip arthroscopy, first described by Burman in 1931 [15], became clinically relevant in the 1990s with advances in instrumentation, fluoroscopy, and joint distraction techniques [16]. The hip joint is divided into two main compartments: the central compartment, encompassing the femoral head, acetabular cartilage, and labrum; and the peripheral compartment, including the femoral neck and surrounding structures. Early arthroscopy was limited to the peripheral compartment due to the absence of distraction. The introduction of fluoroscopic distraction in the mid-1980s enabled separation of the femoral head from the acetabulum, allowing therapeutic interventions in the central compartment [17]. Arthroscopy can be performed using either an inside-out or outside-in approach, determined by the sequence and technique of portal creation.

Inside-Out Technique

The inside-out technique, developed earlier and still widely utilized, begins with the application of axial traction to achieve joint distraction. A spinal needle is introduced under fluoroscopic guidance, typically through the anterolateral (AL) portal, to gain access to the central compartment. Subsequent cannulation and guidewire placement allow for visualization and the establishment of secondary working portals. This method facilitates early access to central compartment structures, including the labrum and acetabular cartilage, and is considered particularly advantageous in cases requiring labral repair or chondral work. However, it demands precise control of distraction to minimize the risk of labral or cartilage injury [18].

Outside-In Technique

**Figure 8:**
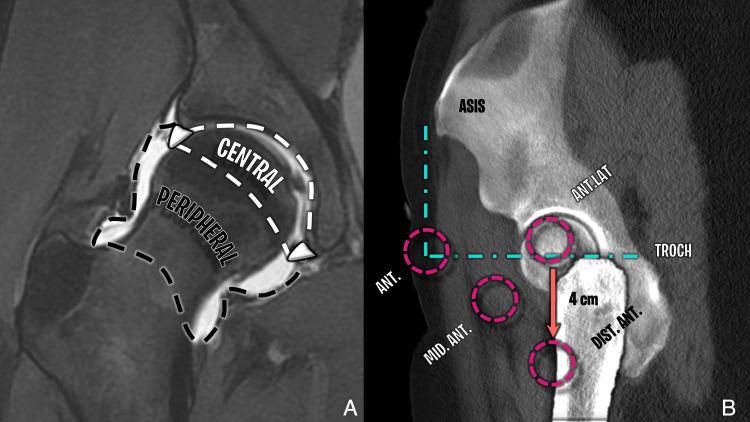
Hip joint compartments and arthroscopic portals (A) Coronal T1-weighted fat-saturated arthrogram showing central (white dotted line) and peripheral (black dotted line) compartments. Articular surfaces are only visible with traction. (B) Reformatted sagittal CT with portal overlays showing common outside-in arthroscopic portals. Image Credit: Juan Pablo Muñoz, corresponding author ASIS: anterior superior iliac spine; ANT: anterior portal; ANT.LAT: anterolateral portal; MID.ANT: mid-anterior portal; DIST.ANT: distal anterior portal

**Figure 9:**
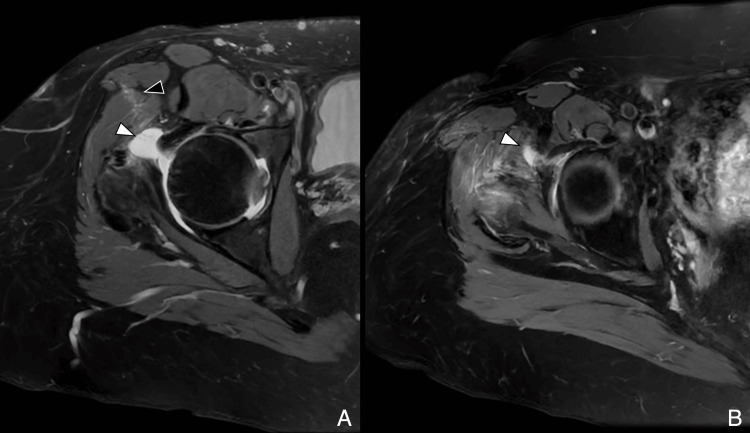
Outside-in arthroscopy (early postoperative MRI) Axial PDFS images (A) at the femoral head level and (B) above the greater trochanter show a trans-gluteal portal with TFL penetration (black arrow) and capsular lobulation at the suture plane (white arrows). Anterolateral portal path varies due to fluoroscopic placement. Image Credit: Juan Pablo Muñoz, corresponding author PDFS: proton density fat-saturated; TFL: tensor fasciae latae

**Figure 10:**
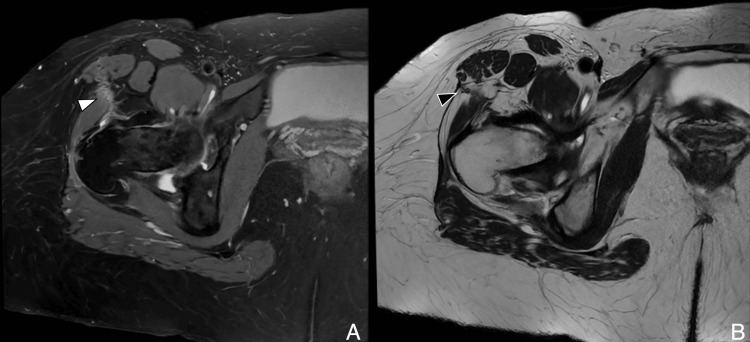
Outside-in arthroscopy (portal tract evolution) (A) Early postoperative axial PDFS image shows anterolateral portal tract as high signal through TFL and gluteus medius, extending to the joint capsule (white arrowhead). (B) Late postoperative axial T2-weighted MRI shows tract replaced by hypointense scar tissue (black arrowhead). Image Credit: Juan Pablo Muñoz, corresponding author PDFS: proton density fat-saturated; TFL: tensor fasciae latae

The outside-in technique targets the peripheral compartment first, minimizing reliance on distraction. Initial portal placement, usually at the AL portal near the tip of the greater trochanter, is guided by anatomical landmarks and fluoroscopy. Working portals, such as the peritrochanteric space portal (PSP) and distal anterolateral accessory portal (DALA), are established distally along the femur. A modified mid-anterior (MMA) portal is often added, located approximately 1.5 cm above and midway between the AL and DALA portals (Figure 8) [19]. This approach provides controlled access to the peripheral compartment, facilitates femoroplasty procedures for cam lesions, and reduces the risk of iatrogenic damage to central compartment structures. Nonetheless, variations in portal trajectories can dissect different muscular or intermuscular planes, which may be evident on postoperative MRI, aiding in the identification of expected soft tissue changes or inadvertent nerve involvement (Figure 9, Figure 10).

Arthroscopy has revolutionized the management of femoroacetabular impingement (FAI), a major indication for the procedure. Techniques such as labral repair, labral reconstruction, cam osteoplasty, pincer rim trimming, and capsular closure are now routinely performed arthroscopically, with high rates of pain relief and return to sport [20,21]. Recent advancements in imaging, including three-dimensional CT and intraoperative fluoroscopy, have enhanced preoperative planning and intraoperative precision [22].

Emerging technologies are poised to further optimize outcomes. Robotic-assisted systems are being introduced to improve the accuracy of resection and portal placement [23], and biological therapies such as platelet-rich plasma (PRP) and stem cell injections are being explored to enhance labral and cartilage healing postoperatively [24]. Furthermore, artificial intelligence and augmented reality navigation are under investigation to support surgical planning and execution [25].

While arthroscopy offers clear benefits, including faster recovery and preservation of native hip anatomy, a steep learning curve persists. Long-term durability appears promising in selected patients, although careful patient selection based on factors such as cartilage status, age, and activity level remains critical to optimizing outcomes [26,27].

A summary of hip surgical approaches is listed in Table 1.

**Table 1:** Summary of common hip surgical approaches PSIS: posterior superior iliac spine; TFL: tensor fascia lata; LFCN: lateral femoral cutaneous nerve; AL: anterolateral; DALA: distal anterolateral

Approach	Access Interval/Technique	Key Features	Common Nerve Risks	Clinical Pros/Cons
Posterior	Incision from inferior trochanter to PSIS; splits gluteus maximus; tenotomy of short rotators	Spares abductors; may show piriformis atrophy	Sciatic nerve (esp. peroneal branch)	Low dislocation risk; good acetabular access; higher nerve risk
Lateral	Splits gluteus medius to expose minimus and joint capsule	Identifiable abductor detachment on imaging	Superior gluteal nerve	Good visualization; risk of abductor insufficiency
Anterior (Smith-Peterson)	Between sartorius and TFL (Smith-Peterson interval)	May spare or detach rectus femoris	LFCN, femoral nerve	Lower dislocation rate; early mobilization
Anterolateral (Modified)	Between TFL and gluteus medius (Watson-Jones interval)	Muscle-sparing versions show minimal scar	LFCN (variable)	Minimally invasive; harder to detect access path on imaging
Hip Arthroscopy (Inside-Out)	Distraction then anterolateral portal for joint access	Direct access to central compartment	Sciatic, LFCN, pudendal	Better central visualization; risk of labral injury
Hip Arthroscopy (Outside-In)	Access starts at peripheral compartment; AL+DALA portals	Reduces central iatrogenic injury	LFCN, muscular branches (portal tract)	Safer for peripheral work; fluoroscopy-dependent

Access-related complications

Nerve Injuries

**Figure 11:**
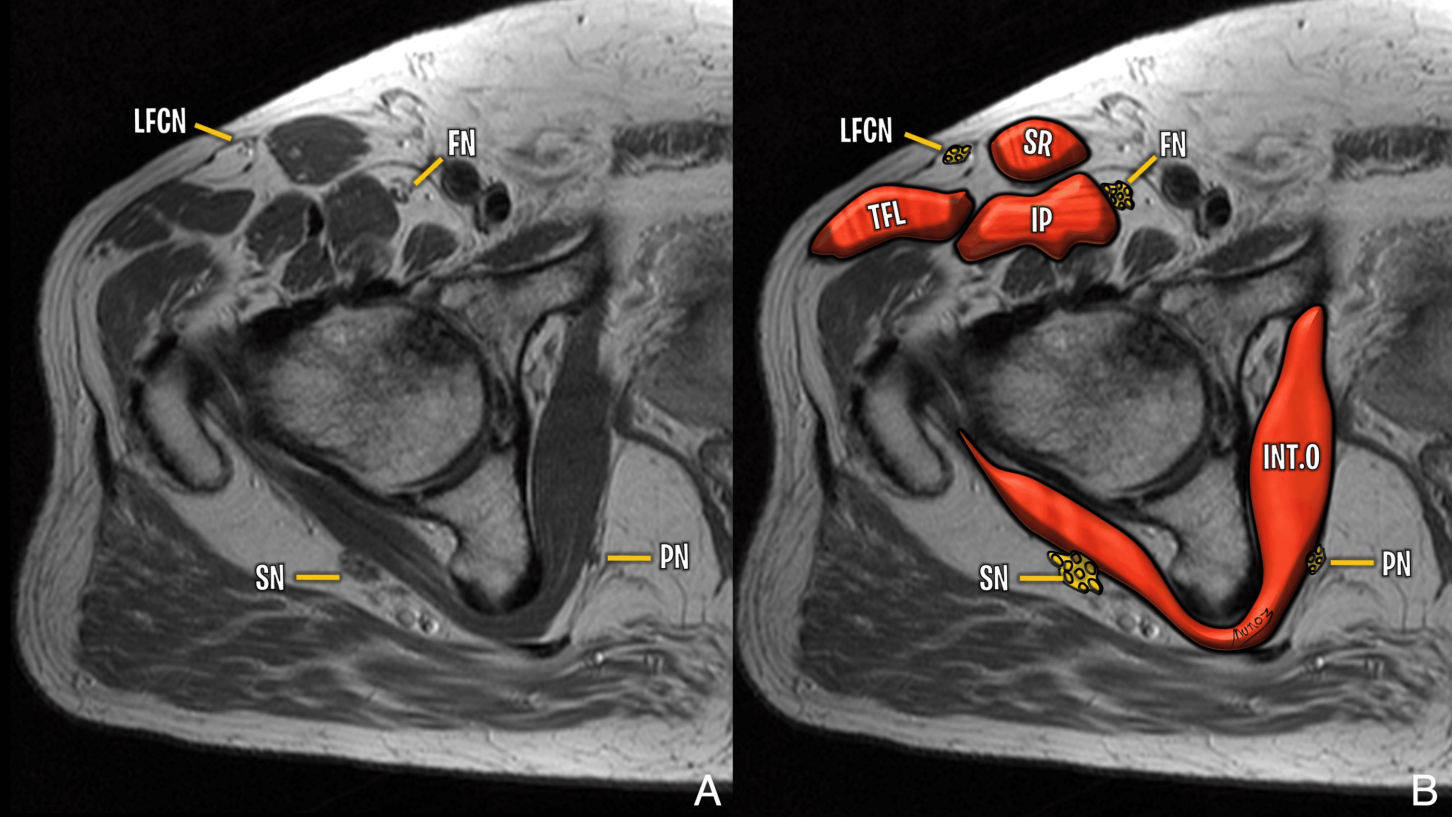
Nerve anatomy around the hip (greater trochanter level) Superior gluteal nerve shown in Figure 3. Image Credit: Juan Pablo Muñoz, corresponding author LFCN: lateral femoral cutaneous nerve; FN: femoral nerve; SN: sciatic nerve; PN: pudendal nerve; TFL: tensor fascia lata; SR: sartorius; INT.O: obturator internus; IP: iliopsoas

Nerve injury is a recognized complication of hip surgery, with an incidence ranging from 0.6% to 3.7% following THA, primarily due to traction, compression, ischemia, or direct transection (Figure 11) [28-30]. Transection injuries are typically caused by sharp dissection, electrocautery, or hardware placement [31].

Each surgical approach carries distinct neural risks. The anterior approach is associated with a high rate of lateral femoral cutaneous nerve (LFCN) neuropathy, reported in up to 81% of cases, and occasional femoral nerve injury (0.8%) [32,33]. The lateral approach carries a 77% incidence of subclinical superior gluteal nerve injury [34]. The posterior approach has a 0.3-2.1% incidence of sciatic nerve injury, predominantly affecting the peroneal branch [35].

Recent large cohort analyses identified revision surgery, female sex, and higher comorbidity burden (Elixhauser Comorbidity Index ≥3) as independent risk factors for perioperative nerve injury after THA [36]. Broader meta-analyses confirm that spinal conditions, postoperative anemia, and hypothyroidism also significantly increase the risk [37]. In hip arthroscopy, nerve complications account for approximately 52.8% of reported adverse events, with traction-related sciatic and pudendal neurapraxia being the most common, while portal-related LFCN injuries are also frequent [38].

**Figure 12:**
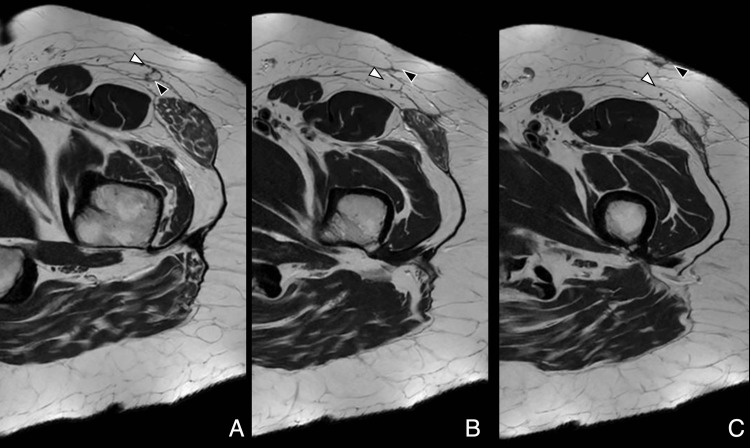
Normal anteromedial portal scar adjacent to LFCN Axial T2-weighted images, proximal to distal, showing linear scar tissue (black arrowhead) along the anteromedial portal tract adjacent to the intact LFCN (white arrowhead). No disruption noted. Patient was asymptomatic. Image Credit: Juan Pablo Muñoz, corresponding author LFCN: lateral femoral cutaneous nerve

**Figure 13:**
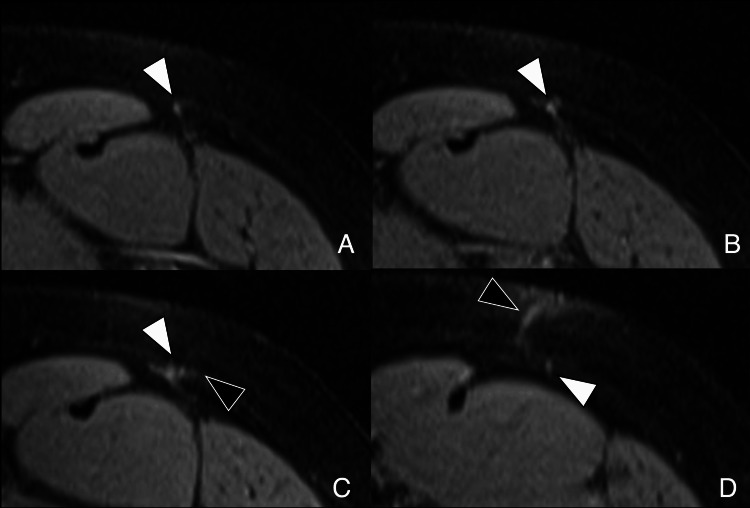
LFCN neuropathy due to portal injury Axial PD fat-saturated images, proximal to distal, showing scar (black arrowhead) contacting the LFCN (white arrowhead) with nerve thickening and high signal. Patient reported dysesthesia over the lateral thigh. Compare the LFCN signal and diameter between slices. Image Credit: Juan Pablo Muñoz, corresponding author LFCN: lateral femoral cutaneous nerve; PD: proton density

**Figure 14:**
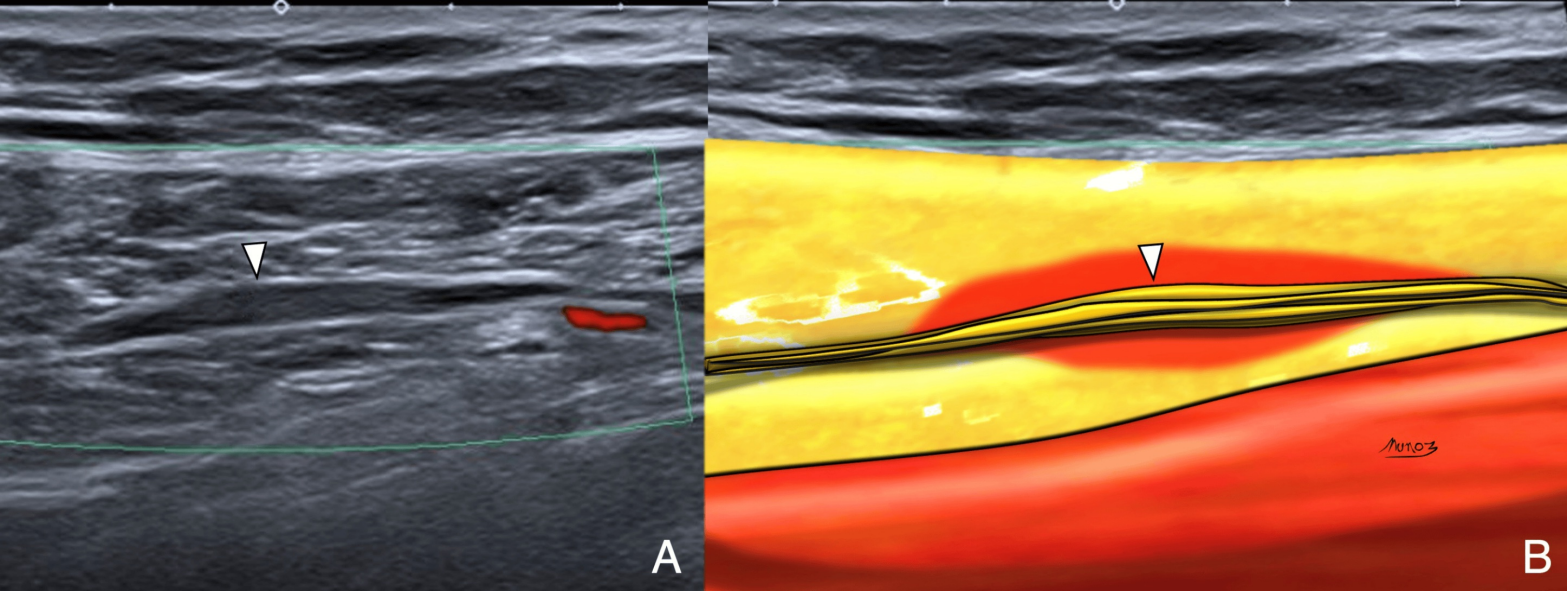
LFCN neuropathy with meralgia paresthetica symptoms. (A) Longitudinal ultrasound with power Doppler shows segmental thickening of the LFCN (white arrowhead). (B) Corresponding diagram. No vascular flow noted, supporting neural origin. Image Credit: Juan Pablo Muñoz, corresponding author LFCN: lateral femoral cutaneous nerve

**Figure 15:**
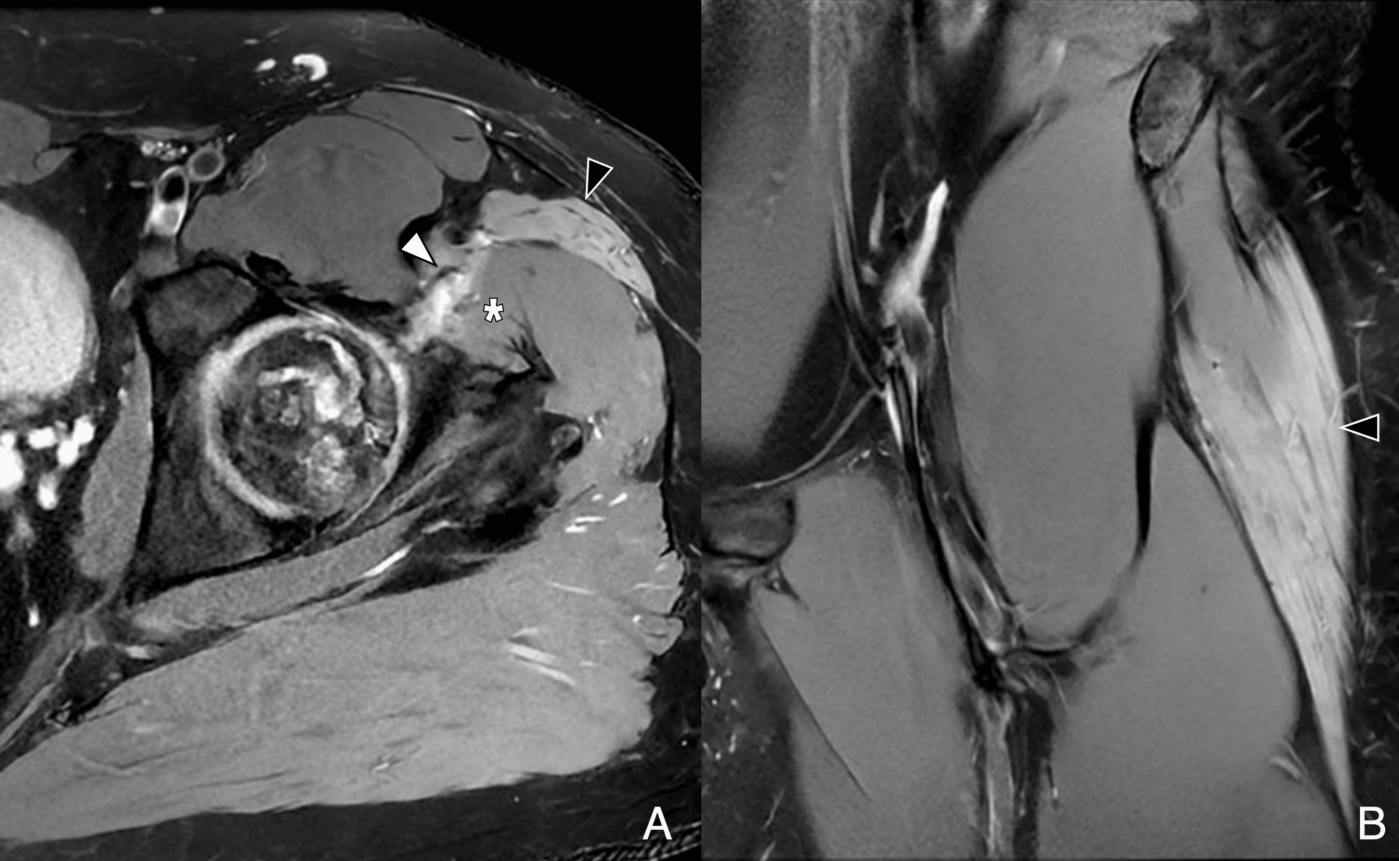
Tensor fascia lata denervation after arthroscopy (A) Postoperative axial PDFS image following outside-in arthroscopy shows a portal access scar traversing the TFL (black arrowhead) and gluteus medius (white asterisk), with diffuse TFL edema consistent with subacute denervation secondary to superior gluteal nerve branch injury. (B) Coronal PDFS image demonstrates full-thickness TFL involvement (black arrowhead), characteristic of denervation. Image Credit: Juan Pablo Muñoz, corresponding author PDFS: proton density fat-saturated; TFL: tensor fascia lata

On imaging, nerve injuries can manifest as direct disruption of nerve continuity (rarely visible on conventional MRI) and indirect signs, such as denervation edema in acute/subacute stages or fatty atrophy of innervated muscles in chronic stages (Figure 12, Figure 13, Figure 14, Figure 15) [37].

Capsular Dehiscence/Insufficiency

The role of capsular repair in hip arthroscopy has gained increasing recognition. Routine closure, particularly following interportal and T-capsulotomies, has been associated with improved functional outcomes, reduced risk of revision surgery, and lower rates of conversion to THA [39-41]. While minimal capsular violation techniques (periportal and puncture capsulotomies) often do not require closure in patients without hyperlaxity [42], larger capsulotomies disrupt key stabilizing structures, especially the iliofemoral ligament, and may result in iatrogenic instability if unrepaired [43].

**Figure 16:**
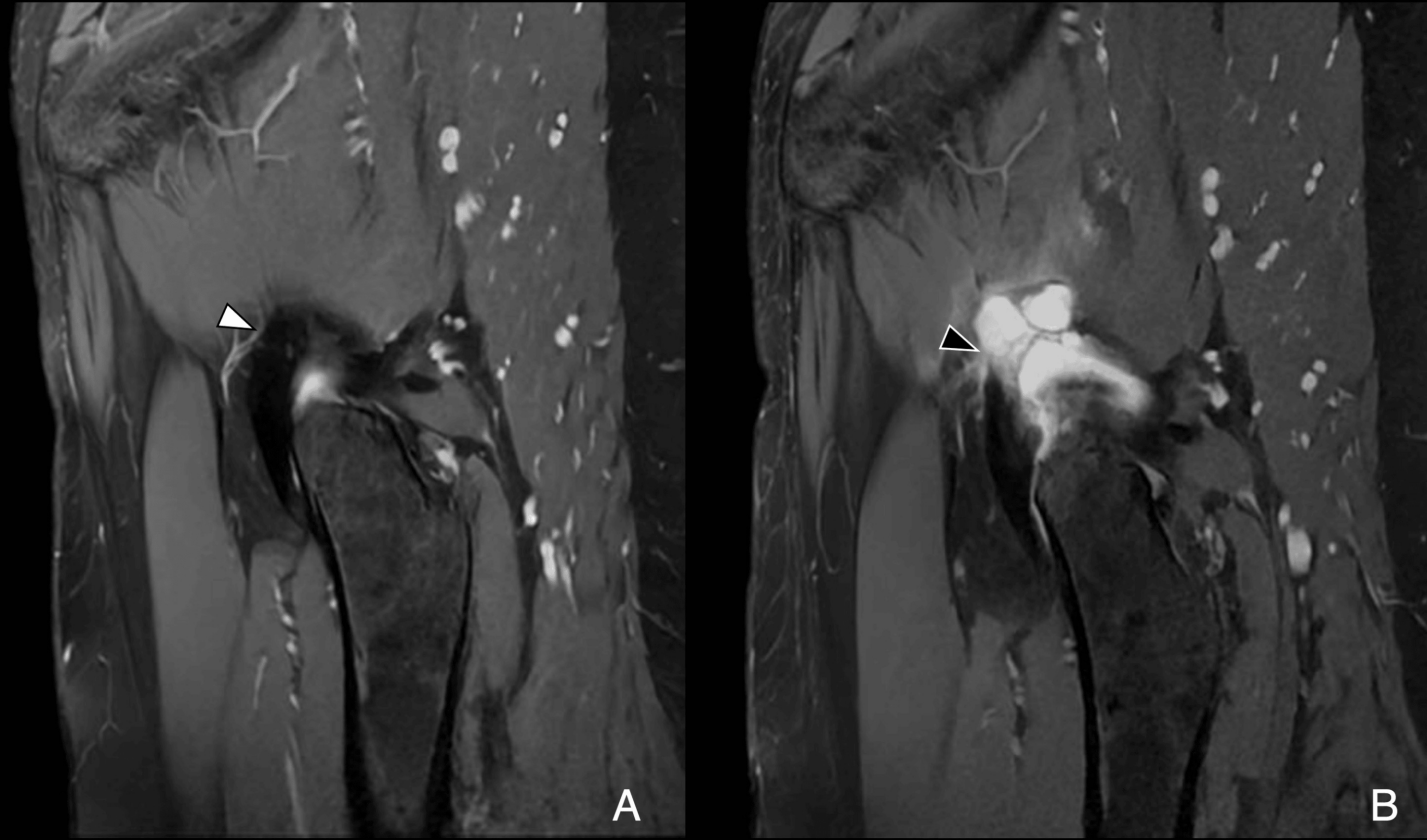
Capsular dehiscence after arthroscopy (A) Sagittal PDFS preoperative image shows a thin, intact capsule (white arrowhead). (B) One-year postoperative image shows lobulated contour at the suture site, consistent with dehiscence (black arrowhead). Image Credit: Juan Pablo Muñoz, corresponding author PDFS: proton density fat-saturated

Capsulorrhaphy usually results in minimal scarring and may be radiologically inapparent. However, postoperative MRI can reveal signs of capsular laxity, hypertrophy, or dehiscence, with bulging or lobulation at the capsular repair site, often visualized on fluid-sensitive sequences (Figure 16) [42-44].

In cases of significant capsular incompetence after arthroscopy, revision surgery with isolated capsular repair can significantly improve outcomes [42]. If tissue is insufficient for repair, capsular reconstruction using iliotibial band autograft or Achilles tendon allograft may be required to restore stability [42,45]. Although capsular reconstruction improves resistance to distraction and rotational instability compared to complete capsulectomy, it does not fully restore native biomechanics [45].

Abductor Insufficiency

Abductor insufficiency is a known complication primarily associated with the lateral approach, where gluteus medius splitting or gluteus minimus tenotomy is performed. Persistent gluteal tendon defects and fatty infiltration, whether from denervation, tendon failure, or iatrogenic injury, are major contributors to dysfunction, potentially leading to abductor syndrome [46].

Imaging findings critical for diagnosis include full-thickness or partial tendon tears, muscle atrophy, fatty infiltration, often graded using Goutallier classifications [47].

**Figure 17:**
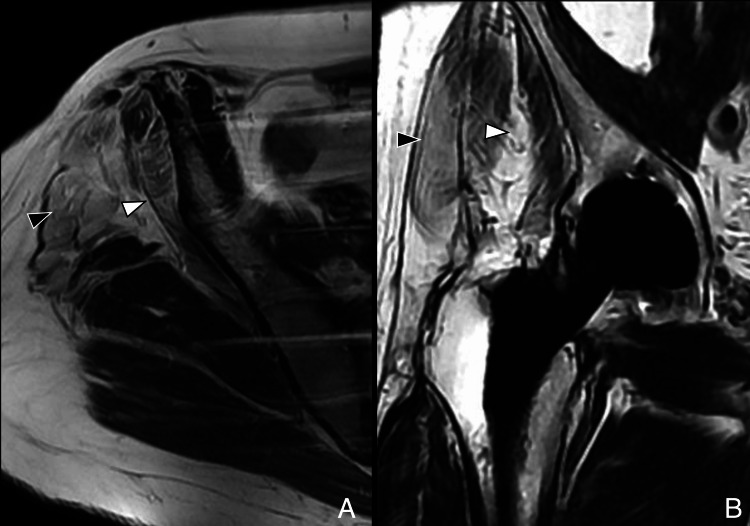
Abductor insufficiency (A) Axial T2-weighted MRI showing diffuse gluteus minimus atrophy (white arrowhead) and anterior gluteus medius atrophy (black arrowhead). (B) Coronal MAVRIC sequence in another patient shows gluteus medius (black arrowhead) and gluteus minimus (white arrowhead) atrophy following lateral approach. Image Credit: Juan Pablo Muñoz, corresponding author MAVRIC: multi-acquisition variable resonance image combination

Comparative MRI studies have confirmed a strong correlation between postoperative tendon defects, advanced fatty degeneration, and symptomatic THA failure (Figure 17) [46,48]. In cases of irreparable abductor deficiency, gluteus maximus tendon transfer has emerged as an effective salvage option, improving pain and function, although residual gait abnormalities (e.g., Trendelenburg sign) may persist [49].

It remains crucial to distinguish pre-existing degenerative changes from postoperative injuries; thus, preoperative imaging comparison is strongly advised wherever available.

New techniques, perspectives, and advances in hip surgical approaches

Recent years have seen significant technological and conceptual advances in hip surgery aimed at improving outcomes, minimizing invasiveness, and enhancing recovery periods.

**Figure 18:**
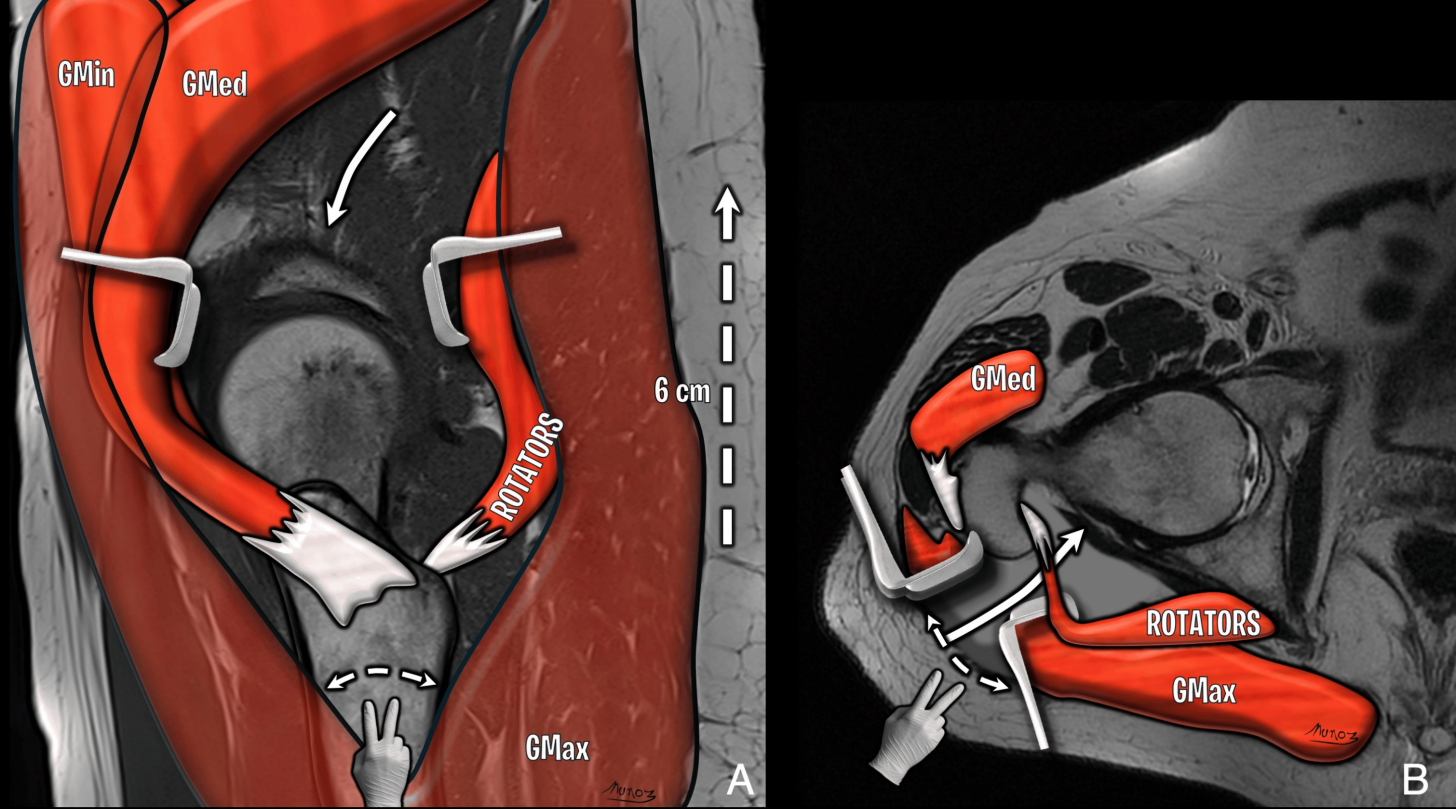
SuperPATH hybrid technique (A) Sagittal T1 and (B) Axial T2 diagrams depicting skin incision posterior to the greater trochanter (white dashed arrow), gluteus maximus (GMax) blunt dissection, gluteus minimus (GMin) and medius (GMed) anterior retraction and rotators posterior retraction; superior access to the joint capsule (white arrow). Image Credit: Original artwork by Juan Pablo Muñoz, corresponding author

Minimally invasive techniques continue to evolve, providing viable alternatives to traditional THA approaches. These include the already mentioned DAA, minimally invasive anterolateral approach (MIS-ALA), minimally invasive posterior approach (MIS-PA), the supercapsular (SuperCap) approach, which accesses the hip capsule from above, and the percutaneously-assisted total hip (PATH) approach, which accesses the capsule from behind and slightly below, all without increasing complication rates [50-53]. Among these, the hybrid SuperPATH technique, combining elements of SuperCap and PATH, stands out for in situ broaching (without dislocation) and its preservation of soft tissues, including complete sparing of the external rotators and capsule preservation, thereby promoting faster postoperative recovery (Figure 18) [53].

Simultaneously, hip arthroscopy for femoroacetabular impingement (FAI) has matured significantly. Surgical strategies focusing on labral repair and reconstruction [54-56], cam and pincer resection [57,58], and capsular management [43] have become essential to restoring hip joint mechanics and preventing instability. Capsular closure, previously overlooked, is now recognized as crucial to improving long-term joint stability and patient outcomes [43].

**Figure 19:**
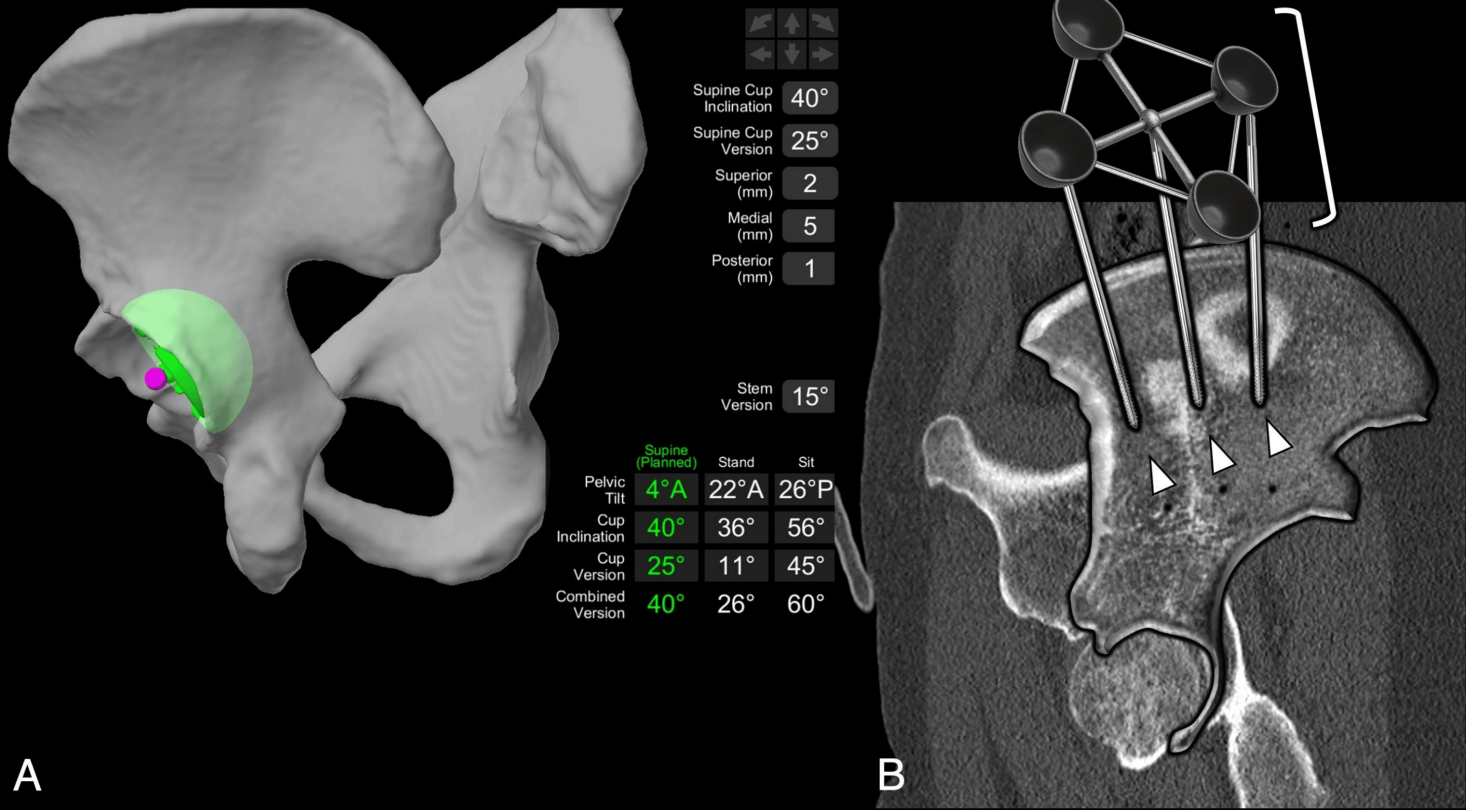
MAKO Robotic-Arm Assisted Surgery System (Stryker, Mahwah, NJ, USA) Preoperative CT-based planning. (A) Standing and sitting pelvic tilt, cup inclination, and version are loaded into the navigation system to ensure proper placement. (B) Pelvic array (reformatted and superimposed sagittal CT slices): three iliac pins (white arrowheads), placed posterior to the ASIS, anchor the pelvic array (white bracket) in place to provide spatial orientation for acetabular reaming, regardless of the patient's position. Image Credit: Juan Pablo Muñoz, corresponding author ASIS: anterior superior iliac spine

Technological innovations are reshaping the landscape. Robotic-assisted hip procedures, using systems such as the MAKO Robotic-Arm Assisted Surgery System (Stryker, Mahwah, New Jersey, United States) (Figure 19) and the NAVIO Surgical System (Smith+Nephew, London, United Kingdom), enable real-time navigation, precise bone resection, and improved implant alignment [59,60]. Parallel advances in imaging, notably 3D CT reconstructions and high-resolution intraoperative fluoroscopy, enhance preoperative planning and intraoperative lesion targeting [61].

Biologic therapies, including PRP and stem cell treatments, are under investigation for their potential to improve cartilage and labral healing [62,63]. Early evidence suggests that these regenerative strategies may accelerate recovery and improve tissue quality postoperatively.

Looking ahead, artificial intelligence is poised to revolutionize hip surgery through predictive modeling, personalized surgical planning, and real-time intraoperative decision support. Future developments also emphasize personalized medicine, incorporating patient-specific imaging and genetic data to tailor interventions [20].

Despite these advances, challenges persist. The technical demands of minimally invasive and arthroscopic techniques impose a steep learning curve, which can affect early outcomes [64]. Furthermore, although short- and medium-term outcomes are promising, the durability of newer techniques in younger and athletic populations requires further long-term evaluation.

## Conclusions

Hip surgical techniques continue to evolve, with variability in adoption and technical execution across institutions. A structured understanding of common surgical approaches and their expected postoperative imaging appearances is essential not only for radiologists but for all clinicians involved in hip surgery follow-up. Recognizing normal surgical changes, identifying complications, and integrating surgical context into imaging interpretation enhances diagnostic accuracy and improves multidisciplinary communication. This knowledge is critical for optimizing patient outcomes and guiding further management.
